# The human HELQ helicase and XRN2 exoribonuclease cooperate in R-loop resolution

**DOI:** 10.1098/rsob.240112

**Published:** 2025-02-19

**Authors:** J. M. Pan, H. Betts, A. Cubbon, L. He, E. L. Bolt, P. Soultanas

**Affiliations:** ^1^ Biodiscovery Institute, School of Chemistry, University of Nottingham, University Park, Nottingham NG7 2RD, UK; ^2^ School of Life Sciences, University of Nottingham, University Park, Nottingham NG7 2RD, UK

**Keywords:** HELQ helicase, XRN2 exoribonuclease, R-loops, RPA, DNA repair

## Abstract

The human HELQ helicase is a superfamily 2, 3′-5 helicase homologous to POLQ and RNA helicases of the Ski2-like subfamily. It is involved in diverse aspects of DNA repair and is an emerging prognosis biomarker and novel drug target for cancer therapy. HELQ interacts with RPA through its inherently disordered N-HELQ domain and hence is recruited to RPA-bound DNA substrates. Our study reveals a novel role for HELQ in R-loop resolution. We show in cells and *in vitro* that HELQ is recruited by RPA at R-loops, which are then resolved if HELQ is catalytically active as an ATPase/helicase. Furthermore, we identify a functional interaction of HELQ with XRN2, a nuclear 5′ to 3′ exoribonuclease, which we suggest coordinates R-loop unwinding by HELQ with RNA digestion by XRN2. Collectively, we assign a new biological function for HELQ in genome stability in metazoans through its involvement with XRN2 in R-loop metabolism.

**Figure:**
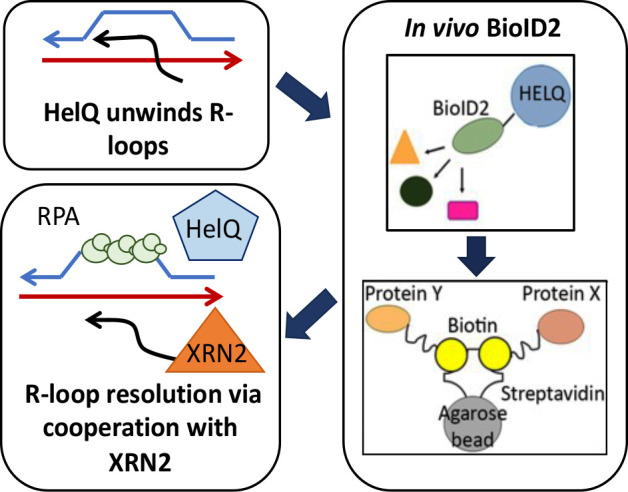

## 1. Introduction

R-loops (RNA–DNA duplexes that displace ssDNA loops) are crucial for regulating gene expression, chromatin organization, class-switch recombination mitochondrial DNA replication, telomere maintenance and DNA repair [1–3]. However, their unresolved accumulation destabilizes replisome and transcription complexes triggering genome instability [1,4–7]. To safeguard against this, all species utilize helicase/annealase proteins to dissociate RNA–DNA duplexes and promote re-annealing of the DNA strands, providing cellular management of R-loop homeostasis [8].

HELQ, a superfamily 2 (SF2) helicase, and its closest homologue the Ski2-like helicase Hel308 contribute to genome stability during DNA repair and homologous recombination in metazoans and archaea, respectively [9–16]. HELQ is a ssDNA-stimulated ATPase that translocates DNA with 3′ to 5′ polarity [17,18] and in addition, it anneals DNA strands in reactions that do not need ATP hydrolysis [10,19]. A non-catalytic N-terminal PWI-like domain of HELQ (N-HELQ) mediates a physical interaction with RPA to load the catalytic C-terminal domain (C-HELQ) to DNA substrates and activate its ssDNA-dependent, ATP-fuelled 3′ to 5′ translocation along the ssDNA substrate [20]. More specifically, the N-HELQ interaction with RPA disrupts the interaction of RPA with ssDNA causing its dissociation from ssDNA and allowing the C-HELQ to bind once the RPA is evicted [20]. In the absence of an interaction with RPA, C-HELQ still exhibits *in vitro* 3′ to 5′ translocase activity along ssDNA [20] through non-specific ssDNA-binding but in the absence of an interaction with RPA HELQ is unlikely to be directed efficiently to the correct genome sites where it is needed *in vivo*. The annealing activity is modulated by RPA and stimulated by the DNA polymerase delta POLD3 subunit [10,19,20]. The interactions of HELQ with RPA and POLD3 likely control DNA repair by homologous recombination in metazoans, alongside further interactions of HELQ with the recombinase Rad51 and its paralogues [21–23].

RPA is a critical sensor of R-loops, as the ‘first responder’ to ssDNA that is displaced by the RNA–DNA hybridization within R-loops. It physically interacts with RNase H1, recruiting it to R-loops where it can degrade RNA to promote their resolution [24,25]. However, in the cell, RNase H1 is mainly localized in mitochondria [26] while RNase H2 is specifically expressed in the G2/M phase of the cell cycle [27]. It is, therefore, likely that other RNases are directly involved in the digestion of the RNAs released from the resolution of R-loops in the cell nucleus when DNA replication is active during the S phase of the cell cycle.

Here, we present *in vitro* and in cells data to show that the interaction of human RPA with HELQ recruits HELQ to R-loops triggering R-loop resolution. This newly identified function of HELQ provides further context to its multiple roles as a hub for controlling replicative DNA repair processes. Furthermore, we show that HELQ functionally interacts with XRN2, a nuclear 5′ to 3′ exoribonuclease that is involved in RNA metabolism, including R-loop resolution [28,29], which digests the RNA released from R-loops during unwinding by HELQ. This suggests a cooperative mode of action between HELQ and XRN2 through recruitment by RPA to R-loops to help maintain genome stability in human cells.

## 2. Material and methods

### 2.1. HELQ and BioID2-HELQ DNA constructs

HELQ cloned into pcDNA3.1 (pAC36) prepared using GeneArt (Thermo Fisher) was used as the template to PCR amplify a HELQ fragment for in-frame fusion with BioID2 in the Addgene plasmid 92308, using JMP-1: 5′-ATCCGAGCTCGGTACCAAGCGCTAGCGCCATGGCTTGG-3′ and JMP-2: 5′-GGCTGATCAGCGGTTTAAACGGTACCTCATCATGCTTTGTCAG-3′.

#### 2.1.1. BioID2 pulldowns in human U2OS cells

Osteosarcoma (U2OS) cells were cultured at 80% confluency in 6 cm dishes and transfected with pcDNA3.1 BioID2-HelQ or BioID2 backbone pcDNA3.1 using TransIT-X2^®^ Dynamic Delivery System (Mirus, MIR6004). After 72 h, cells were harvested and confirmed for BioID2-HELQ protein expression using western blots. For BioID2-HELQ pulldowns growth media were supplemented with 50 μM of Biotin and incubated for 18 h prior to cell lysis. The U2OS cell lysate preparation was adapted from [30]. Briefly, cells were collected using cell scrapers and lysed as detailed in the original protocol. Crude protein lysate was incubated with 200 μl of Gelatin conjugated Sepharose 4B (GE Healthcare, 17095601), for 4 h at 4°C with constant rotation. Following incubation, the beads were spun down for 3 min at 800 r.p.m. and the supernatant was collected. The clarified cell lysate was then incubated with 50 μl of Streptavidin Sepharose High-Performance Beads (GE Healthcare, 17511301) at 4°C overnight with constant rotation. After overnight incubation, the beads were spun down for 3 min at 800 r.p.m. and washed with 0.5 ml of PBS three times, then resuspended in dH_2_O, flash-frozen and stored at −80°C for subsequent analysis.

### 2.2. Identification of BioID2-HELQ interactors

Biotinylated proteins recovered from cells by immobilization on beads were reduced (100 mM DTT), alkylated (120 mM iodoacetamide) and digested with trypsin (Promega, V5111) overnight at 37°C. The supernatant was loaded into automated liquid chromatography–mass spectrometry (LC-MS/MS) analysis. All LC-MS/MS experiments were performed using the commercially available service by the University of Cambridge using a Dionex Ultimate 3000 RSLC nano-UPLC system and a Q Exactive Orbitrap mass spectrometer.

Data were processed using Protein Discoverer (version 2.3, Thermo Fisher). Briefly, all MS/MS data were submitted to the Mascot search algorithm (Matrix Science, version 2.6.0) and searched against a common contaminants database (125 sequences; 41 129 residues) and the UniProt human database (93 609 sequences; 37 041 084 residues). Scaffold (version 4.10.0, Proteome Software Inc.) was used to validate MS/MS-based peptide and protein identifications. Peptide identifications were accepted at greater than 95.0% probability by the Scaffold Local FDR algorithm and 99.0% probability with at least two identified peptides. Protein probabilities were assigned by the Protein Prophet algorithm [31]. Similar peptides that could not be differentiated based on MS/MS analysis alone were grouped to satisfy the principles of parsimony while proteins sharing significant peptide evidence were grouped into clusters. A *t*‐test was used to compare significance (*p* < 0.05) of difference between the two groups.

### 2.3. Western blots and antibodies

Transfected cells were treated with 200 μg ml^−1^ of G418 and reseeded in 10 cm culture dishes; three dishes per plasmid were cultured to 80% confluency. At 16 h before harvest, the media were supplemented with Biotin (final concentration 50 ng ml^−1^), cells were harvested on ice and resuspended in lysis buffer (8 M urea in 50 mM Tris pH 7.4 with 1× protease inhibitor (Halt Protease Inhibitor Cocktail, EDTA-Free, Thermo Scientific, 87785); 100 mM AEBSF·HCl (4-(2-aminoethyl)benzenesulfonyl fluoride hydrochloride), 80 μM aprotinin, 5 mM bestatin, 1.5 mM E-64, 2 mM leupeptin, 1 mM pepstatin A and 1 mM DTT). Cell lysate was incubated on ice for 30 min, DNAse I was added and incubated at room temperature for 10 min. Then, the cell lysate was centrifuged at 12 000 r.p.m. for 10 min at 4°C. Twenty-five percent of the cell lysate was collected for western blot, samples were resolved by SDS-PAGE through a 4–15% gradient gel and transferred onto PVDF membrane (0.22 µm; Thermo Fisher, 88520). All antibodies were diluted in 5% (w/v) BSA in TBS-Tween 20 (0.05% v/v). Primary antibody polyclonal rabbit anti-HelQ was purchased from Genosphere Biotechnologies specifically generated against purified N-HelQ and used at 1:10 000 dilution. Mouse anti-actin (Santa Cruiz, sc-8432) was used at 1:1000 dilution and rabbit anti-Myc (Abcam, ab32) was used at 1:1000 dilution.

### 2.4. Purification of XRN2, XRN2(D54A), HELQ, RPA and GFP-RNase H (D210N)

XRN2 plasmid (pJMP1/pRSETA-XRN2) was generated using the Invitrogen GeneArt gene synthesis service from the sequence at Uniprot (Gene code: Q9H0D6) and codon-optimized for protein expression in *Escherichia coli*. To generate 6xHis-MBP tagged and TEV-cleavable, XRN2 (6xHis-MBP-TEV-XRN2) primers containing AflII and BamHI for PCR assembly into pET28-MBP-TEV-6xHis (Addgene 69929): F-MBP-XRN2: 5′-TGAAAATCTATACTTCCAAGGATCCATGGGTGTTCCGGCATTTTTCC-3′ and R-MBP-XRN2: 5′-CACAATTTCATGGTGTCCACTTAATTCCAATTATAACGACCGCTAG-3′.

RNase inactive XRN2 (6xHis-MBP-TEV-XRN2(D54A)) [32] was generated using site-directed mutagenesis primers: F-F1-D54A mut: 5′-GCCGCGCGGCAGCCATATGGCTAGCGGTAAAATCGAAG-3′, R-F1-D54A mut: 5′-TAATGCCGTTCATTGCCAGGTACAGGTTATC-3′, F-F2-D54A mut: 5′-CCTGGCAATGAACGGCATTATTCATCCG-3′ and R-F2-D54A mut: 5′-AGTGGTGGTGGTGGTGGTGCTTAATTCCAATTATAACGACCG-3′.

Protein production used chemically competent *E. coli* BL21-AI transformed with XRN2 plasmid that grows in carbenicillin (50 µg ml^−1^) supplemented 2xYT medium at 37°C for 3 h or until 0.6 OD_600_. Protein overexpression was induced using 0.02% (w/v) l-arabinose and 1 mM IPTG and cultured at 18°C for 16−18 h. Liquid cultures were centrifuged at 4000*×g* for 20 min at 4°C and biomass was resuspended in resuspension buffer (10% (v/v) glycerol, 50 mM Tris pH 7.4, 20 mM imidazole, 50 mM NaCl and 2.5 mM DTT) supplemented with complete™ ULTRA tablets, EDTA-free, glass vials Protease Inhibitor Cocktail and then stored at −80°C if not lysed and used immediately.

XRN2 and XRN2(D54A) expressing biomass was lysed using sonication on ice providing lysate that was clarified at 45 000×*g*, 4°C for 30 min. The supernatant was collected and filtered through a 0.45 µm syringe filter (Minisart^®^ Syringe Filter, polyethersulfone (PES), pore size 0.45 µm). 6xHis-MBP-XRN2 and 6xHis-MBP-XRN2(D54A) were purified using the tandem 6xHis-MBP affinity tags through an ÄKTA Start system. The clarified lysate was loaded into a Hi-Trap chelating HP column (GE Healthcare) in buffer A (10% (v/v) glycerol, 50 mM Tris pH 7.4, 20 mM imidazole, 50 mM NaCl and 2.5 mM DTT) and eluted with the same buffer in a gradient of increasing imidazole concentration to 1 M. Fractions corresponding to a UV absorption peak (280 nm) were analysed using SDS-PAGE, with desired fractions pooled and loaded into an MBP Trap HP column pre-equilibrated with buffer MBP-A (10% (v/v) glycerol, 50 mM NaCl, 50 mM Tris–HCl pH 7.4 and 2.5 mM DTT) and eluted in the same buffer in a gradient of increasing maltose to 10 mM. Pooled fractions were treated with TEV protease to remove the His-MBP tags and proteins analysed using SDS-PAGE. Pooled protein fractions were dialysed into 50 mM Tris pH 7.4, 50 mM NaCl, 10% (v/v) glycerol and 2.5 mM DTT overnight at 4°C and concentrated to 1−2 ml using Vivaspin^®^ 20, 3000 MWCO PES (VS2091). Protein concentration was measured using a NanoDrop™ Lite spectrophotometer (ND-LITE-PR) measuring the absorbance at 280 nm and validated using a Pierce™ BCA Protein Assay Kit (23225).

HELQ and RPA trimer proteins were expressed and purified as described previously [20,33]. Protein concentrations were measured using a NanoDrop™ Lite spectrophotometer (ND-LITE-PR) measuring the absorbance at 280 nm and validated using a Pierce™ BCA Protein Assay Kit (23225) following manufacturer’s instructions. All purified proteins were aliquoted, flash-frozen and stored at −80°C.

GFP-RNase H (D210N) was overexpressed from Addgene plasmid 174448 using *E. coli* BL21 AI (Invitrogen). Cells were grown in carbenicillin containing 2xYT broth at 37°C with shaking until 0.6 OD_600_. Protein overexpression was induced by adding IPTG (1 mM) and l-arabinose (0.02% w/v) for 16 h at 18°C. Cells were harvested through centrifugation, suspended in buffer A (50 mM Tris–HCl pH 7.5, 500 mM NaCl, 20 mM imidazole and 10% (v/v) glycerol) and lysed by sonication. Soluble cell extract clarified by centrifugation was loaded into a HisTrap HP 5 ml affinity column (Cytiva) pre-equilibrated with buffer A. Bound proteins were eluted in a linear imidazole gradient of 20 mM−1 M. Pooled fractions were loaded into a gel filtration HiLoad 16/600 Superdex 200 pg column (GE Healthcare) in 50 mM Tris–HCl pH 7.5, 500 mM NaCl, 1 mM DTT and 10% (v/v) glycerol. GFP-RNase H (D210N) containing fractions were pooled, spin concentrated and flash frozen for storage at −80°C. Protein concentrations were calculated using BCA assay (Thermo Fisher).

### 2.5. Generation of M13 dsDNA R-loops

*In vitro* plasmid R-loops were generated with CRISPR-Cas interference reactions [34]. Briefly, 50 ng of M13 dsDNA (rf DNA, NEB, N4018) was incubated with 100 nM of Cascade complex comprising a crRNA targeting M13 DNA, in Helicase Buffer (20 mM Tris–HCl pH 8.0, 1 mM DTT, 100 mM NaCl and 0.1 mg ml^−1^ BSA) in 10 µl and incubated at 37°C for 30 min. Formation of R-loops by Cascade and the effects of HELQ and/or RPA on R-loops were assessed with agarose gel electrophoresis. Equimolar concentrations of HELQ and RPA (200 nM) were incubated with M13 DNA-based R-loops in Helicase Buffer at 37°C for 10 min. Reactions were stopped by the addition of 2 µg of proteinase K and STOP solution (2.5% (w/v) SDS and 200 µM EDTA), and samples were resolved through 0.8% (w/v) TAE agarose gels at 10 V for 2 h using a Mini-Sub GT Cell (BioRad). Then gels were stained with ethidium bromide for imaging by a UV transilluminator.

### 2.6. Generation of short synthetic R-loops

For helicase assays, synthetic oligonucleotides were used to generate *in vitro* R-loops by annealing two DNA and one Cy5-labelled RNA oligonucleotides as follows.

Top DNA strand (RL):

5′-GGGTGAACCTGCAGGTGGGCGGCTGCTCATCGTAGGTTAGTTGGTAGAATTCGGCAGCGTC-3′

Bottom DNA strand (RL):

5′-GACGCTGCCGAATTCTACCAGTGCCTTGCTAGGACATCTTTGCCCACCTGCAGGTTCACCC-3′

Cy5 labelled RNA strand (RL):

5′-AAAGAUGUCCUAGCAAGGCAC-Cy5-3′

For the forked-R-loop, the following synthetic oligonucleotides were used.

Top DNA strand with ssDNA overhang (RL):

5′-CAACGTCATAGACGATTACATTGCTAGGGTGAACCTGCAGGTGGGCGGCTGCTCATCGTAGGTTAGTTGGTAGAATTCGGCAGCGTC-3′

Bottom DNA strand (RL):

5′-GACGCTGCCGAATTCTACCAGTGCCTTGCTAGGACATCTTTGCCCACCTGCAGGTTCACCC-3′

Cy5-labelled RNA strand (RL):

5′-AAAGAUGUCCUAGCAAGGCAC-Cy5-3′

To form the structures shown in the results, the constituent oligonucleotides were mixed in 1:1.2 ratio of labelled oligonucleotide : unlabelled oligonucleotide (5 µM : 6 µM) in annealing buffer (10 mM Tris–HCl pH 7.5, 1 mM NaCl, 1 mM EDTA and 200 mM potassium acetate) to a final volume of 50 μl and heated at 95°C for 10 min before cooling to room temperature over hours. Oligonucleotides were transferred to ice for immediate use or frozen in aliquots. R-loops were visualized by PAGE and confirmed by RNase H degradation (25 nM of the R-loop was incubated with 1:20 RNase H in RNase H buffer (NEB, M0297S)).

### 2.7. HELQ helicase and XRN2 exoribonuclease assays

HELQ helicase assays in the presence and absence of RPA were carried out using synthetic R-loops (25 nM) and in Helicase Buffer (20 mM Tris–HCl pH 8.0, 1 mM DTT, 100 mM NaCl, 10 mM MgCl_2_, 5 mM ATP (or AMPPNP) and 0.1 mg ml^−1^ BSA) to make 20 µl reaction volume. Samples were incubated at 37°C before terminated by STOP solution (200 µM EDTA, 2 µg ml^−1^ proteinase K and 2.5% (w/v) SDS). Orange G (80% (v/v) glycerol and 2 mg ml^−1^ Orange G powder) was added to each reaction and all samples were then loaded onto a 10% (w/v) polyacrylamide TBE gel with images provided using a Typhoon Amersham (Cytiva).

XRN2 exoribonuclease assays used the same buffer and stop conditions as helicase assays but forked-R-loop substrate (25 nM) and addition of XRN2 (250 nM), XRN2(D54A) (250 nM), HELQ (100 nM) and RPA (100 nM) proteins at 37°C. Reaction products were then loaded onto a 12% TBE–urea gel and resolved for 2 h for imaging on a Typhoon scanner (Cytiva). Image quantification was carried out using ImageJ [35], and data analysis was carried out with GraphPad Prism software (version 8.00 for Windows, GraphPad Software, La Jolla, California, USA).

### 2.8. R-loop detection by *in vitro* dot blot assays

Dot blot assays were adapted from a method in [36]. Briefly, R-loops were aliquoted (1–2 μl) onto Hybond-N membranes and air-dried for 30 min. Nucleotides were crosslinked to the membrane using 240 mJ of UV light (254 nm) before incubation in blocking buffer; TBS-Tween 20 (0.05% v/v) with 5% (w/v) milk for 1 h at room temperature. R-loops were detected using S9.6 antibody (1:1000) diluted in blocking buffer at 4°C, followed by probing with secondary antibody (1:4000 goat anti-mouse IgG, alkaline–phosphatase conjugated) and imaging using NBT/BCIP solution in visible light for quantitative analysis and densitometry. High-quality dot blot digital images were quantified using ImageJ [35]. Images were converted to an 8-bit image from their original format and the colours inverted for clearer imaging. Each dot on the membrane was encapsulated with the circular drawing tool and the integrated density values measured and plotted using GraphPad Prism software (version 8.00 for Windows, GraphPad Software, La Jolla, California, USA).

### 2.9. Human cell culture

Osteosarcoma (U2OS) cells were used in BioID2 pulldown experiments and RKO (colon carcinoma) cells were used for studying R-loops in cells. Engineered RKO cell lines were sourced from Nanna Therapeutics using CRISPR-Cas9 technology (electronic supplementary material, figure S1). All cells were incubated in humidified incubators at 37°C with 5% CO_2_. RKO cell lines were cultured in Rosewell Memorial Institute 1640 medium (RPMI) supplemented with 10% (v/v) heat-inactivated fetal bovine serum (FBS, Sigma Aldrich), 100 units ml^−1^ of streptomycin and 100 µg ml^−1^ of penicillin and passaged when cells reached 80% confluency to a new flask.

### 2.10. In cells, R-loop detection using GFP-RNase H (D210N)

R-loop detection using GFP-RNase H (D210N) was a modification from a protocol in [37]. RKO cells were seeded into 8-well chamber slides and treated with 2 mM thymidine for 16 h, recovered for 8 h and then synchronized for 16 h; media containing thymidine were removed and replaced with fresh media and cells were allowed to recover for 8 h. After recovery, 2 mM of thymidine was added for further 16 h to synchronize cells at the onset of the S phase. Media were removed and cells washed with PBS twice and those were fixed in 100% (v/v) methanol at −20°C for 5 min. Cells washed twice with PBS were permeabilized in PBS–Triton X (0.25% v/v) at room temperature for 20 min and washed again with PBS and incubated for 3 h at 37°C with gentle shaking, with 1:50 diluted RNase H in RNase H buffer (NEB, M0297S). Cells were then washed with PBS containing Tween 20 (0.05% v/v) once for 5 min, followed by PBS wash for 5 min and treated with GFP-RNase H (D210N), diluted 1:100 in RNase H buffer, at 37°C for 2 h with gentle shaking. Cells were subsequently washed once with PBS for 5 min at room temperature, coverslips mounted using Antifade mounting with DAPI (P36931) and stored at 4°C for imaging. Images were taken by a Leica TCS SP8 SMD, at 40× magnification and quantified using FiJi. Statistical analyses used GraphPad Prism 8.0 where R-loops in *HELQ+/+* cells were the baseline for comparison with *HELQ−/−* and *HELQ D463A* cells.

### 2.11. Cell transfections for complementation

*HELQ−/*− cells were transfected with pcDNA3.1*HELQ*, generated using GeneArt gene synthesis (Thermo Fisher) by providing a codon-optimized sequence of human HELQ cloned into the pcDNA3.1 backbone. The pcDNA3.1*HELQ* plasmid was prepared using Qiagen plasmid Maxi kit and quantified using Nanodrop 200 (Thermo Fisher). Cells were transfected in each eight-well chamber slide with pcDNA3.1*HELQ* (50 ng) and FuGene transfection reagent (Promega) mixed at a 1:2 molar ratio, respectively, and then incubated for 15 min at room temperature. The mixture was then added dropwise to the media and incubated for 72 h before fixing, as described above.

## 3. Results

### 3.1. RPA recruits human HELQ to R-loops and the ATPase/helicase activity of HELQ is essential for R-loop resolution

**Figure 1.**
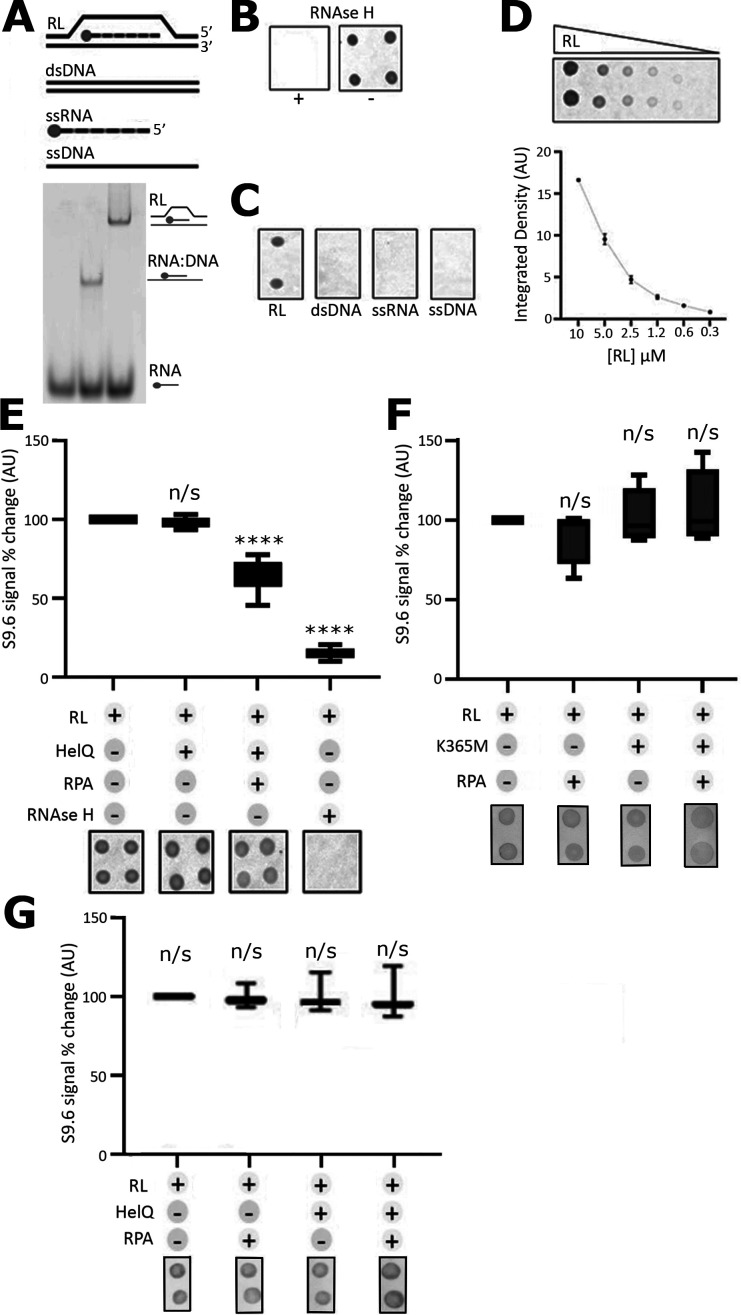
Synthetic oligos can be used to generate *in vitro* R-loops that are detectable through the S9.6 antibody and gel electrophoresis. (A) Schematic representation of the synthetic oligonucleotide substrates, RL (R-loop), dsDNA, ssRNA and ssDNA, used in the dot blot experiments (top panel). The RNA was fluorescently labelled at the 3’-end with the Cy5 fluorophore (indicated by a filled circle). The sequences of the synthetic oligonucleotides are shown in §2. Formation of R-loops was verified by PAGE (bottom panel). (B) To further validate the generation *in vitro* R-loops, RNase H degradation assays were carried out and products were probed with dot blots using the S9.6 antibody (1:50 diluted in RNase H buffer (NEB, M0297S)). (C) The specificity of S9.6 for R-loops *in vitro* compared with dsDNA, ssRNA and ssDNA was verified by dot blots. (D) The S9.6 signal (±s.e., *n* = 3) detected and quantified by dot blots was dependent on the concentration of R-loops, although this relationship was not linear. Lack of linearity is likely due to incomplete binding of oligonucleotides to the membrane leading to uneven loss of substrate during washing steps. (E) Treatment of R-loops with HELQ did not affect R-loops (unpaired two-tailed Student’s *t*‐test showed n/s, *p* = 0.6850) but in the presence of HELQ and RPA the S9.6 signal decreased significantly (****p < 0.0001)*,* suggesting that HELQ resolves R-loops in the presence of RPA. Treatment with RNase H completely abolished the S9.6 signal confirming the identity of R-loops (****p < 0.0001). Error bars represent ±s.e. from three independent experiments (*n* = 3 with four repeats per experiment). (F) A Walker A mutation (K365M) completely abolishes the HELQ ability to resolve R-loops in either the presence or absence of RPA (n/s *p* = 5933 and n/s *p* = 0.6648, respectively) whereas RPA on its own does not significantly affect R-loops (n/s *p* = 0.3266). Error bars represent ±s.e. from three independent experiments (*n* = 3 with two repeats per experiment). (G) Control reactions with R-loops in the presence of RPA alone, HELQ alone and in the presence of RPA with AMPPNP replacing ATP in the helicase reactions. Unpaired two-tailed Student’s *t*-tests show that there are no significant (n/s) changes in the R-loops in these conditions (RPA alone *p* = 0.8596, HELQ alone *p* = 0.8410 and RPA+HELQ *p* = 0.9532). Error bars represent ±s.e. from three independent experiments (*n* = 3 with two repeats per experiment). Selective dot blots are shown below the graphs in (*E)* and (*G)* for visual illustration. An example of a complete dot blot experiment can be seen in electronic supplementary material, figure S2.

The association of human RPA with R loops and subsequent recruitment of RNase H1 is a key event in their resolution [24,38]. We have shown previously that RPA interacts with HELQ and recruits it to DNA substrates [20]. Given the similarity of HELQ to the Ski2-like DExH-box RNA helicases, we hypothesized that HELQ may be recruited to R-loops by RPA to resolve them by unwinding the RNA and annealing the DNA strands. In initial experiments, we used a rapid dot blot assay to investigate this hypothesis (figure 1 and electronic supplementary material, figure S2A). A synthetic R-loop was assembled from two complementary ssDNA strands and one ssRNA strand that was complementary to a sequence in one of the ssDNA strands (figure 1A). R-loops were confirmed by PAGE and dot blot assays using S9.6 anti-RNA:DNA antibody and RNase H treatment (figure 1A,B). RNase H digestion of the RNA in R-loops is an established method to verify R-loops [39] while the S9.6 antibody is often used for detection of R-loops [40,41]. We confirmed the specificity of S9.6 for R-loops over dsDNA, ssRNA and ssDNA in this assay and that the R-loops were lost after incubation with RNase H which degrades the RNA (figure 1B,C). The S9.6 signal we detected and quantified in the dot blot assays was dependent on the R-loop concentration (figure 1D). The specificity of S9.6 to report R-loops *in vivo* has been disputed [41], but we observed that it was highly specific for R-loops over dsDNA, ssDNA and ssRNA in the *in vitro* dot blot assay. Incubation of synthetic R-loops with HELQ produced marginal statistically insignificant reduction of S9.6 signal, but incubation of R-loops with HELQ after addition of RPA resulted in significant reduction of S9.6 signal, indicating resolution of R-loops (figure 1E). Furthermore, the S9.6 signal was dependent on protein (HELQ and RPA) concentrations (electronic supplementary material, figure S2BC). The ATPase/helicase activity of HELQ appears to be essential for R-loop resolution, as incubation with a Walker A K365M HELQ mutant, lacking in ATPase/helicase activity, in the presence and absence of RPA did not result in reduction of S9.6 signal (figure 1F). Further evidence that the ATPase/helicase activity of HELQ is required for R-loop resolution is provided in control reactions with HELQ, in the presence or absence of RPA and with the non-hydrolysable analogue of ATP, AMPPNP (figure 1G). Replacing ATP with the non-hydrolysable analogue AMPPNP in the reactions resulted in no changes in R-loops, suggesting that ATP hydrolysis by HELQ is essential for R-loop resolution. RPA alone has no detectable effect on R-loops (figure 1G).

**Figure 2.**
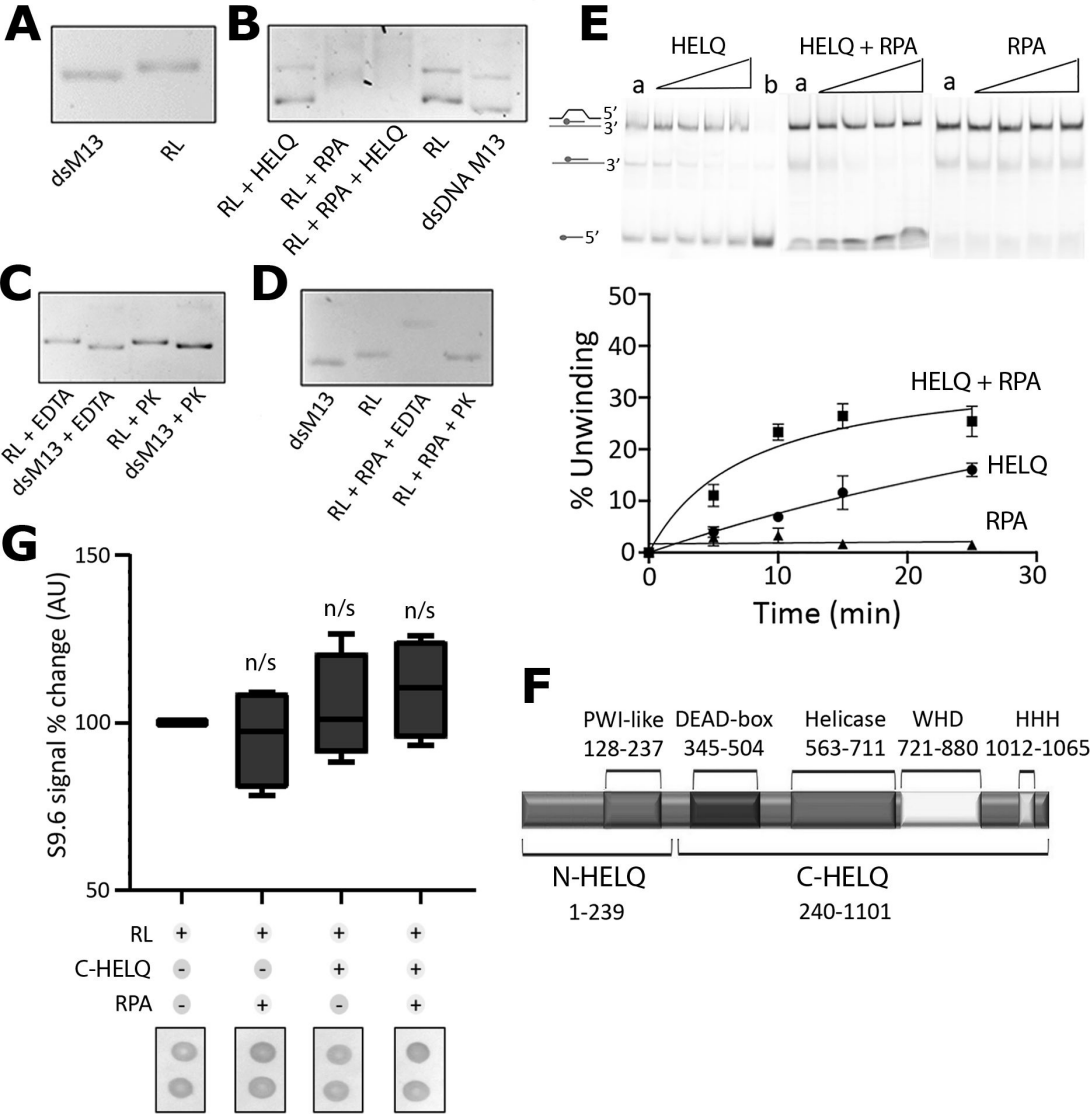
HELQ binds to and resolves synthetic R-loops through interaction with RPA. (A) *In vitro* plasmid based R-loops were generated as previously described [34]. Supercoiled M13 dsDNA plasmid (dsM13) migrated marginally faster compared with R-loop containing supercoiled M13 dsDNA plasmid (RL). (B) Incubations of R-loop containing supercoiled M13 dsDNA (RL) plasmid with HELQ, RPA and RPA+HELQ in the absence of ATP, as indicated. Control incubations of RL and dsM13 without any proteins are also shown. RPA associates with the R-loop and produces smeared shifts characteristic of heterogeneous RPA-RL complexes while in the presence of HELQ these complexes become even more smeared due to increased heterogeneity. (C) Quality control experiments to determine the effect of EDTA and proteinase K (PK), used to remove the cascade complex after the generation of the R-loop, on the stability and electrophoretic mobility of RL and dsM13 plasmids. EDTA and PK have no effect on the stability and electrophoretic mobility of RL and dsM13 plasmids. (D) RPA binding to the R-loop in the presence of EDTA causes significant mobility shift of RL which is reversed when RPA is digested by PK. The substrate reverts to an R-loop showing that mere binding of RPA to the R-loop is not sufficient to resolve it. (E) *In vitro* HELQ helicase assays with a synthetic R-loop show that HELQ can resolve R-loops. The highest level of R-loop resolution was achieved when HELQ and RPA (±s.e., *n* = 3) were incubated with synthetic R-loops, compared with HELQ alone, which showed moderate R-loop resolution activity. RPA had no detectable effect on synthetic R-loops. (F) Schematic representation of the domain architecture of human HELQ. The N-HELQ and C-HELQ domains are shown together with the locations of important structural features; the four-helix bundle of a PWI-like fold, DEAD-box, core helicase, Winged Helix Domain (WHD) and Helix-Hairpin-Helix (HHH) elements. (G) Whisker plots showing that the C-HELQ domain, carrying the helicase core activity but lacking the RPA-interacting N-HELQ domain, is not capable of resolving R-loops. Removal of N-HELQ abrogates the HELQ interaction with RPA which is essential for the removal of R-loops (the error bars represent maximum and minimum values from three independent experiments, *n* = 3 with two repeats per experiment). Unpaired two-tailed Student’s *t*-tests show that there are no significant (n/s) changes in the RL+RPA, RL+C-HELQ and RL+RPA+C-HELQ plots relative to the R-loop (RL) alone plot (*p* = 0.5941, *p* = 0.6226 and *p* = 0.2369, respectively).

To mimic R-loop formation in a more physiological substrate than R-loops constructed from short oligonucleotide substrates, we introduced an R-loop into supercoiled circular M13 dsDNA, generated by a CRISPR-RNA (crRNA) ‘payload’ that is delivered to complementary DNA sequence by the *E. coli* CRISPR immunity effector, as described previously [34]. Once the R-loop formed, the Cascade complex was removed by treatment with proteinase K to leave behind the naked R-loop incorporated into the supercoiled M13 dsDNA. Generation of an R-loop by Cascade targeting supercoiled M13 dsDNA caused a measurable upward shift in its migration as a single band through an agarose gel (figure 2A), as was observed before [34]. When we incubated the R-loop supercoiled M13 dsDNA with HELQ, RPA or HELQ plus RPA in the absence of ATP, we observed distinct effects in its migration through an agarose gel. Addition of HELQ alone resulted in no significant change in the migration of the M13 dsDNA band (figure 2B). RPA alone gave an upward shift of the M13 dsDNA band, including some smearing indicative of dynamically changing, heterogeneous RPA-R-loop complexes. The addition of HELQ and RPA together resulted in further smearing of the RPA-HELQ-R-loop complexes (figure 2B). The formation of nucleoprotein complexes with RPA and HELQ was also confirmed with separate electrophoretic mobility shift assays using a small synthetic R-loop, with RPA and RPA+HELQ showing clear shifts while HELQ on its own did not produce a shift, consistent with what is seen in figure 2B (electronic supplementary material, figure S3). EDTA and proteinase K used to stop and remove the Cascade complex from the M13 dsDNA after the formation of the R-loop had no visible effects on the appearance and migration of the R-loop supercoiled M13 dsDNA through the agarose gel (figure 2C). Proteinase K treatment of the RPA-R-loop complexes resulted in removal of RPA and abolition of the upward band shift through the agarose gel, returning to a position consistent with R-loop migration (figure 2D). Collectively, these data suggest that RPA recruits HELQ at R-loops.

To gain further evidence for RPA-mediated recruitment of HELQ and unwinding of R-loops, we carried out HELQ helicase assays with a synthetic R-loop as substrate in the presence and absence of RPA. In these assays, HELQ alone exhibits a basal helicase activity and unwinds some of the R-loops, but it does so more efficiently in the presence of RPA, with RPA alone control reactions showing no significant R-loop unwinding, as expected (figure 2E). Further control helicase assays with C-HELQ, C-HELQ + RPA, N-HELQ and N-HELQ + RPA showed no R-loop unwinding confirming the importance of having full length HELQ (electronic supplementary material, figure S4). HELQ interacts with RPA70 through the N-HELQ (residues 1−239) that is mostly intrinsically disordered, with the remaining C-HELQ (residues 240−1101) comprising the SF2 helicase domains required for ATPase, DNA translocation and annealing activities (figure 2F) [10,20]. Incubating the synthetic R-loop substrate with C-HELQ in the presence and absence of RPA did not reveal significant reduction in S9.6 signal, consistent with the requirement for the N-HELQ region for the HELQ-RPA interaction and recruitment of HELQ at R-loops (figure 2G).

Collectively the *in vitro* data presented here are consistent with previous studies on the RPA-HELQ interaction [20] and the notion that HELQ is recruited at R-loops through the known direct physical interaction of N-HELQ with RPA to resolve R-loops by unwinding them through the helicase activity of C-HELQ.

### 3.2. Deletion of *HELQ* results in increased R-loop formation in cells

**Figure 3.**
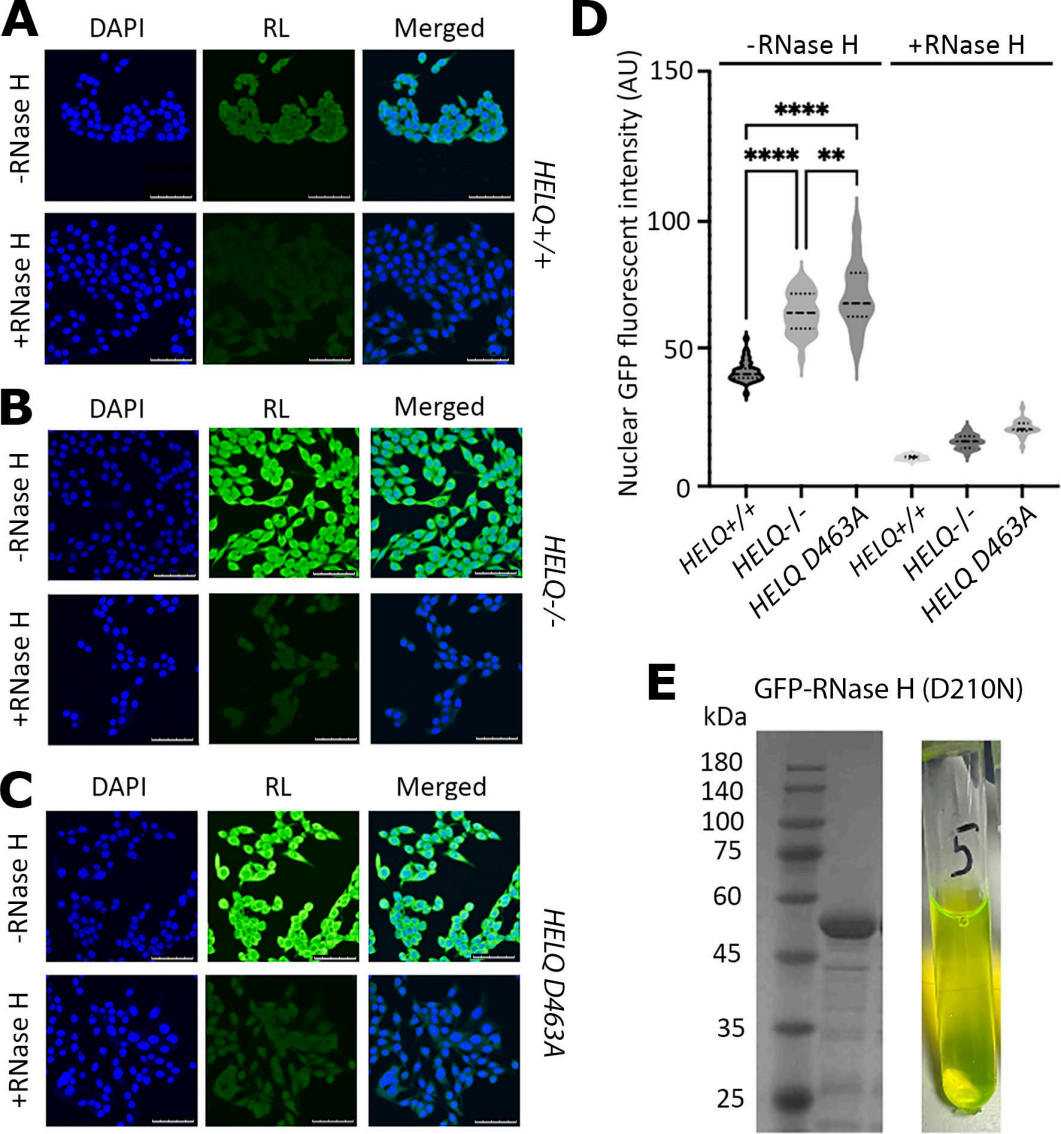
RKO *HELQ−/*− and *HELQ D463A* cells exhibit higher levels of R-loops compared to *HELQ+/ +* cells. (A) Representative fluorescence microscopy of RKO *HELQ+/ +* cells showing images of nuclei stained with DAPI, R-loops stained with GFP-RNase H (D210N) and merged. Fixed cells were either treated (+RNase H) or not treated (−RNase H) with fully active RNase H prior to staining with inactive GFP-RNase (D201N) conjugate. Pre-treatment with RNase H drastically reduces staining confirming the identity of R-loops. White size bars in all images represent 0−75 μm. (B) Representative fluorescence microscopy of RKO *HELQ−/*− cells showing images of nuclei stained with DAPI, R-loops stained with GFP-RNase H (D210N) and merged. Fixed cells were either treated (+RNase H) or not treated (−RNase H) with fully active RNase H prior to staining with inactive GFP-RNase (D201N) conjugate. Pre-treatment with RNase H drastically reduces staining confirming the identity of R-loops. White size bars in all images represent 0−75 μm. (C) Representative fluorescence microscopy of RKO *HELQ D463A* HELQ inactive cells showing images of nuclei stained with DAPI, R-loops stained with GFP-RNase H (D210N) and merged. Fixed cells were either treated (+RNase H) or not treated (−RNase H) with fully active RNase H prior to staining with inactive GFP-RNase (D201N) conjugate. Pre-treatment with RNase H drastically reduces staining confirming the identity of R-loops. White size bars in all images represent 0−75 μm. Higher magnification images equivalent to *(A–*C) can be seen in electronic supplementary material, figure S4. (D) Image quantification was carried out on FiJi, following a protocol previously established [42] and statistical analyses were performed on GraphPad Prism 8.0. Quantification of GFP signal from cells (*n* = 35) showed highest levels of R-loop signal (****p < 0.0001) in *HELQ D463A* cells containing an inactive HELQ, followed by *HELQ−/*− cells lacking the HELQ protein (****p < 0.0001) compared with the *HELQ+/+* cells. Comparison between *HELQ−/*− and *HELQ D463A* cells showed a small but significant accumulation of R-loops in the latter compared with the former (**p < 0.01). (E) SDS-PAGE showing the purified GFP-RNase H (D210N) protein (left panel) and a sample of the purified GFP-RNase H (D210N) protein exhibiting green fluorescence (right panel), as expected.

To investigate whether HELQ plays a role in R-loop resolution in cells, we utilized fluorescence microscopy to detect R-loops in human RKO cells engineered by Nanna Therapeutics using CRISPR-Cas9 technology to have the *HELQ* gene deleted or replaced with a *HELQ* gene carrying the D463A mutation that inactivates the helicase activity of HELQ (electronic supplementary material, figure S1). This is a Walker B motif mutation that inactivates the helicase activity of HELQ [20] and was chosen over the Walker A motif inactivating K365M mutation because it was a better match for gRNA design. We compared fluorescence microscopy images stained for R-loops from *HELQ+/+* cells with *HELQ−/−* and *HELQ D463A* cells. In synchronized RKO cells using thymidine to ensure all cells were stalled at the onset of S-phase (electronic supplementary material, figure S5), *HELQ−/*− cells exhibited increased accumulation of R-loops compared with *HELQ+/+* cells (figure 3A,B and electronic supplementary material, figure S4A,B). Similarly, *HELQ D463A* cells exhibited high levels of R-loops (figure 3A,C and electronic supplementary material, figure S6AC). In fact, statistical analysis showed significant increase of R-loops in *HELQ−/*− cells compared with *HELQ+/+* cells (****p < 0.0001), and *HELQ D463A* cells also exhibited significantly high levels of R-loops compared with *HELQ+/+ cells* (****p < 0.0001) (figure 3D). Comparison between *HELQ−/*− and *HELQ D463A* cells showed a small but significant increase in R-loop accumulation in the latter compared with the former (**p < 0.01).

**Figure 4.**
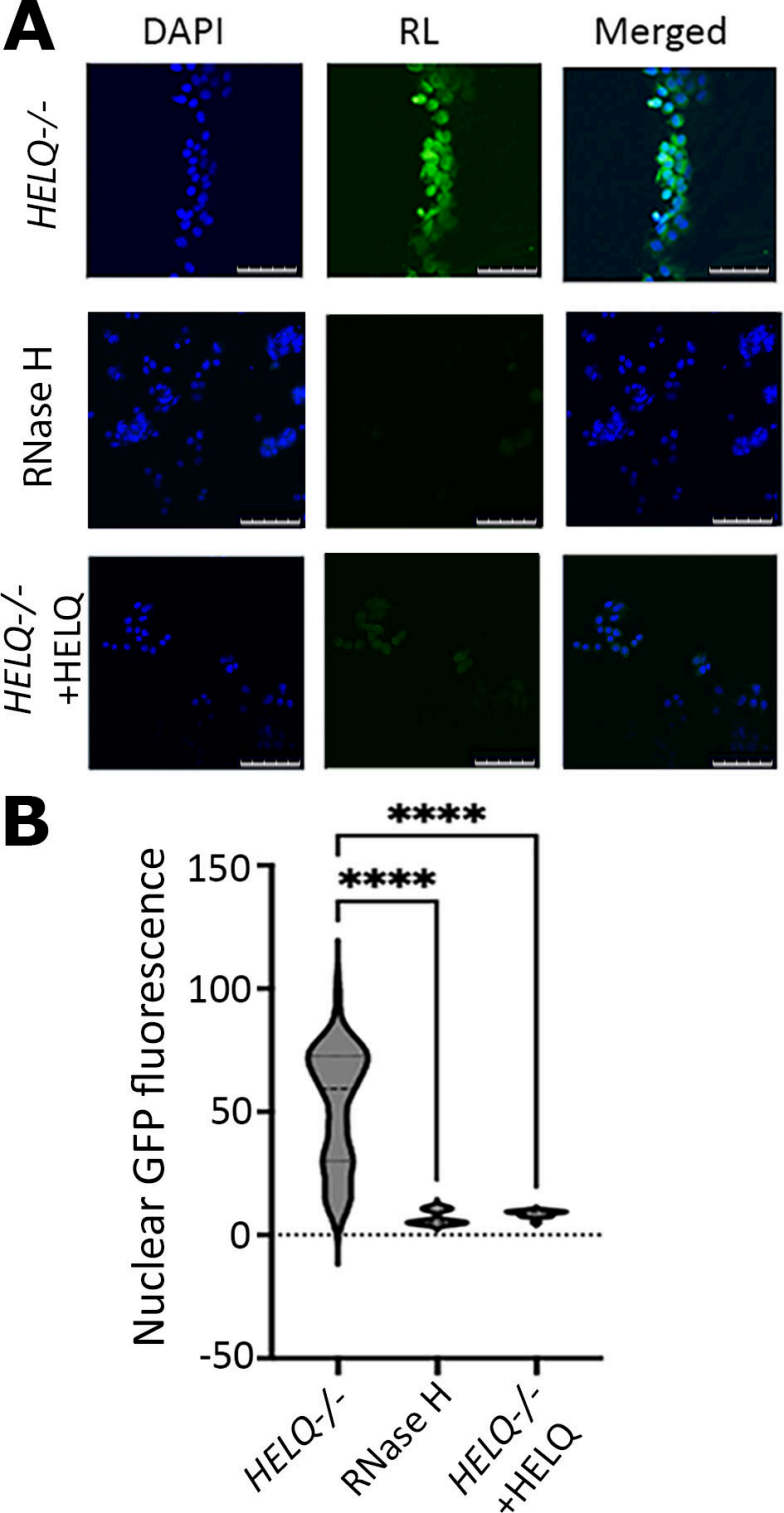
R-loop levels in cells decreased when HELQ was overexpressed in RKO HELQ−/− cells. (A) Representative fluorescence microscopy of *HELQ−/*− cells showing images of nuclei stained with DAPI, R-loops stained with GFP-RNase H (D210N) and merged (top images). Control images of fixed cells treated with active RNase H prior to staining are also shown to confirm that what was stained by the inactive GFP-RNase H (D210N) represented R-loops (middle images). Complementation of *HELQ−/*− cells by transfecting with the pcDNA3.1*HELQ* plasmid and transiently expressing active HELQ for 72 h results in drastic decrease of R-loop levels (bottom images). White size bars in all images represent 0−75 μm. Higher magnification images can be seen in electronic supplementary material, figure S5. (B) Quantification of fluorescence data from complementation experiments shows that overexpression of HELQ in *HELQ−/*− cells significantly reduced R-loop levels (*n* = 35, ****p < 0.0001).

To avoid problems associated with the *in vivo* affinity of the S9.6 antibody for non-R-loop structures containing RNA [43], we used a GFP-RNase H1 conjugate inactivated by a D201N mutation for RNase H catalytic activity in these experiments to stain R-loops in fixed cells for imaging [37] (figure 3E). Pre-treating fixed cells with fully active RNase H drastically reduced staining by the GFP-RNase (D210N) conjugate serving as a control to confirm the identity of R-loops (see +RNase images in figure 3A–C and electronic supplementary material, figure S6A–C). Complementation experiments with transient overexpression of HELQ in *HELQ−/*− cells resulted in marked decrease of R-loop formation (figure 4A,B and electronic supplementary material, figure S7). We conclude that HELQ is involved in R-loop homeostasis *in vivo*, in addition to resolving R-loops *in vitro*, expanding what we know about how HELQ contributes to human DNA repair.

### 3.3. BioID2 pulldowns identify a functional interaction with XRN2 exoribonuclease

**Figure 5.**
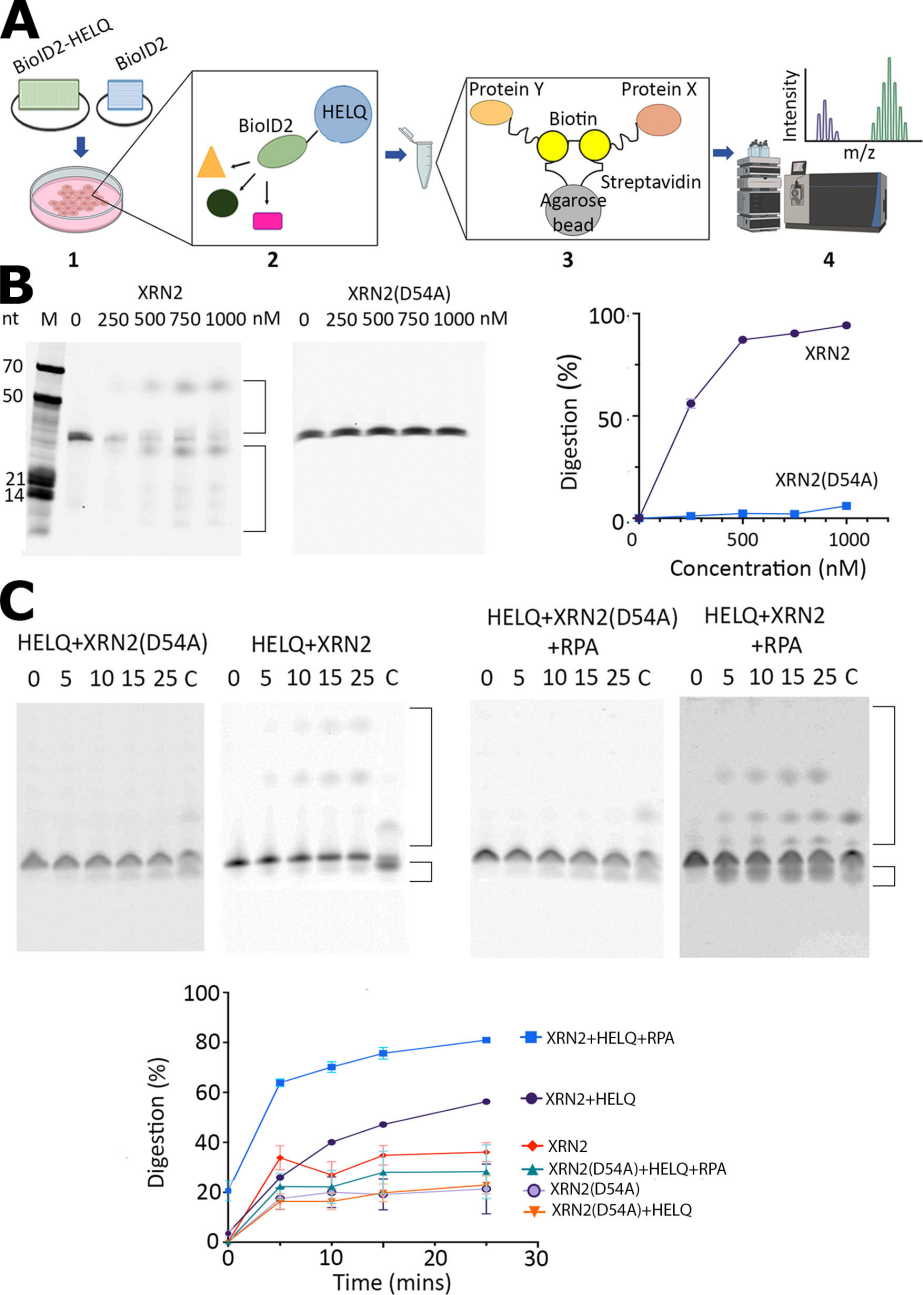
BioID2 pulldowns and XRN2 exoribonuclease assays. (A) BioID2 is a method for screening physiologically relevant protein–protein interactions [44]. **1.** Transfection into cells of the BioID2-tagged HELQ. **2.** Proteins that interact with HELQ are biotinylated *in vivo*. **3.** Biotinylated proteins are pulled down with streptavidin conjugated agarose beads. **4.** Proteins are analysed by LC-MS/MS and identified by peptide fingerprinting from relevant databases. (B) Representative urea–TBE gels to test the exoribonuclease activity of XRN2 and XRN2(D54A) at different concentrations, as indicated using a 34mer RNA Cy5-labelled at the 3′ end (5′-ACUCACUCACUCAAAAGAUGUCCUAGCAAGGCAC-3′-Cy5). Experimental details can be found in §2. Aberrant electrophoresis of the 3′-Cy5 labelled fragments produced by XRN2 digestion is apparent as has been observed before for 5′-Cy5 labelled oligonucleotides [45]. Digestion products are indicated by brackets on the right side of the gels. The graph shows quantification of the gel data confirming the 5′ to 3′ exoribonuclease activity of XRN2 and lack of activity of the XRN2 D54A mutant. (C) Representative gels from time course (0−25 min, as indicated) exoribonuclease assays. These assays were carried out with the forked R-loop substrate incubated with combinations of the HELQ (100 nM), XRN2 (250 nM), XRN2(D54A) (250 nM) and RPA (100 nM) proteins, as indicated. Lanes marked C show control reactions (25 min) with RNase H for comparison. The graph shows quantification of gel data from the exoribonuclease assays. Data were plotted as percent intact RNA substrate remaining at 0, 5, 10, 15 and 25 min (*n* = 2). Error bars show standard errors (±s.e.).

The RNAs released from resolution of R-loops are presumably degraded by ribonucleases but our knowledge of specific ribonucleases that are involved in R-loop resolution is rudimentary. We hypothesized that ribonucleases may cooperate with HELQ in R-loop resolution. To identify potential ribonucleases involved with HELQ in R-loop resolution, we carried out a protein pulldown assay in U2OS cells using a proximity-based labelling approach, namely BioID2. The biotin ligase protein interaction identification generation 2 (BioID2) is a method for screening physiologically relevant protein–protein interactions that occur in living cells. In its simplest form, BioID consists of generating plasmid constructs that express the protein of interest fused to a biotin ligase enzyme (figure 5A) [44]. Generation 2 BioID2 utilizes a smaller more promiscuous biotin ligase from *Aquifex aeolicus*, to enhance biotinylation of interacting or spatially proximal proteins *in vivo* [46]. Using a BioID2-HELQ conjugate, we pulled down several potential HELQ-interactants (electronic supplementary material, figure S8A). Analysis of trypsin generated peptides by LC-MS/MS and database searching identified a potentially interesting exoribonuclease, XRN2 (electronic supplementary material, figure S8B).

We cloned, expressed and purified 6XHis-MBP-XRN2, and constructed a 6xHis-MBP-XRNA2(D54A) ribonuclease inactive mutant, which was also cloned, expressed and purified in an identical manner to the 6XHis-MBP-XRN2 protein (electronic supplementary material, figure S9A). The ribonuclease inactivating XRN2 D54A mutation was identified by structural and sequence comparisons between XRN1 and XRN2. A previously identified ribonuclease inactivating D35A mutation was described in XRN1 [32] and by carrying structural comparisons, using the AlphaFoldDB XRN1 (Uniprot C9JCZ8) and XRN2 (Uniprot Q9H0D6) structural models, and a sequence comparison between XRN1 and XRN2 we identified and constructed the ribonuclease inactivating D54A mutation in XRN2 (see electronic supplementary material, figure S9B). Ribonuclease experiments using a 3′-Cy5 labelled synthetic ssRNA (see §2) confirmed that our purified XRN2 was a 5′ to 3′ exoribonuclease (figure 5B). Exoribonuclease activity from XRN2 in these assays generated several observable products in urea gels, including slowly migrating short 3′-Cy5 labelled RNA fragments, like those observed previously with 5′-Cy5 labelled small DNA oligonucleotides [45]. This unusual electrophoretic mobility of Cy5-labelled oligonucleotides is a consequence of the influence of their mass/charge ratios [45]. As expected, no such products were observed from the XRN2 (D54A) inactive mutant protein even at 1 μM concentration (figure 5B).

To test whether XRN2 cooperates with HELQ in the resolution of R-loops, we constructed a synthetic forked-R-loop using the same 21mer 3′-Cy5 labelled RNA from the helicase assays and appropriate synthetic DNA oligonucleotides (see electronic supplementary material, figure S9C; and §2). This substrate was designed to direct optimal translocation of the 3′ to 5′ HELQ helicase along the bottom strand on which the RNA is annealed to approach the RNA from its 5′-end and make it accessible to the XRN2 5′ to 3′ exoribonuclease activity to gradually digest the RNA towards the 3′-end where the Cy5 label is attached (electronic supplementary material, figure S9C). The forked-R-loop was then used in exoribonuclease assays with various combinations of proteins and products were resolved in TBE–urea gels (figure 5C). In these assays measuring exoribonuclease activity as a function of time, XRN2 activity was stimulated by addition of HELQ and further stimulated by addition of RPA plus HELQ together (figure 5C). Control assays with the XRN2(D54A) inactive mutant protein did not reveal significant exoribonuclease activity in the presence of HELQ or HELQ plus RPA beyond some basal low level non-specific degradation of the RNA over time (figure 5B). These data are consistent with RPA-stimulated R-loop unwinding by HELQ that liberates RNA as substrate for digestion by XRN2.

## 4. Discussion

The SF2 helicase HELQ is emerging as a major factor controlling genome stability involved in several DNA repair pathways, nucleotide excision repair (NER), double strand break repair (DSBR) and inter-strand cross link (ICL) repair, through its interactions with RPA, POLD3, RAD51 and RAD51 paralogues [10,19,21–24,47]. The known physical interaction of HELQ with RPA appears to facilitate HELQ binding to ssDNA by displacing RPA so that HELQ can trigger its ssDNA-dependent helicase/translocase activity and promote annealing of homologous ssDNA regions [20]. Here, we report a new RPA-mediated function for HELQ, which is the resolution of R-loops as shown by *in vitro* and in-cell assays. Dot blot and helicase assays show that HELQ unwinds R-loops more efficiently in the presence of RPA, while knocking out the *HELQ* gene results in significant accumulation of R-loops in *HELQ−/*− compared with *HELQ+/+* cells. Interestingly, introducing the helicase deactivating D463A mutation in the *HELQ* gene results in a further small, but significant increase in the accumulation of R-loops in *HELQ(D463A)* compared with the *HELQ/*− cells (**p < 0.01). A potential explanation for this observation is that RPA-mediated recruitment of the HELQ(D463A) protein at R-loops results in accumulation of this inactive helicase at R-loops thus preventing other helicases from being recruited to process and resolve R-loops. Dysregulated R-loops compromise genome stability mainly by increasing replication–transcription conflicts and promoting single-stranded and double-stranded DNA breaks [1,4–7]. Several helicase/annealase enzymes are involved in R-loop resolution [8] and our in-cell R-loop detection data unequivocally add the HELQ helicase/annealase to the list of helicases involved in R-loop resolution.

RNAs released from R-loops after their unwinding are processed and degraded by various RNA decay pathways. Ribonucleases RNase H1 and H2 have been reported to target and digest RNAs in R-loops *in vitro* [24,25]. However, the sub-cellular localization and expression patterns of RNases H are not consistent with R-loop resolution in the nucleus when DNA replication is active during the S phase of the cell cycle. RNase H1 is mostly localized in nucleoli and in mitochondria [26,48] and cannot be involved in degradation of all RNAs released after the unwinding of R-loops across the nucleus, while RNase H2 is specifically expressed in the G2/M phase of the cell cycle [27] and hence unlikely to be a major ribonuclease involved in removal of R-loops to eliminate genome-destabilizing replication–transcription conflicts during the S phase of the cell cycle when DNA replication is active. By comparison, XRN2 is a 5′ to 3′ exoribonuclease localized in the nucleus [49] and could potentially be one of the nucleases involved in the resolution of nuclear R-loops. XRN2 can resolve RNA:DNA hybrids formed at double strand break sites which allows non-homologous end joining repair to proceed [29], regulates the stability of RNA:DNA R-loops forming at telomeres by degrading the long non-coding telomeric RNA TERRA [50], and resolves RNA:DNA R-loops forming at transcription termination sites of protein coding genes promoting RNAPII removal from the DNA template [51]. Downregulation of XRN2 expression causes R-loop accumulation and DNA damage [28,52], while XRN2 physically interacts with helicases such as Senataxin and DDX5 to degrade RNAs released from RNA:DNA R-loops [52–54]. Consistent with the relevant literature, our data suggest that XRN2 also digests RNAs released from the resolution of nuclear R-loops by HELQ. Previous work established that the RPA-HELQ interaction displaces RPA from DNA such that HELQ can then load and activate its C-HELQ domain with the ssDNA dependent ATPase/helicase activity [20]. Therefore, we propose that RPA, as the first responder to R-loop detection and processing, recruits HELQ at R-loops which then displaces RPA triggering the ssDNA-dependent ATPase activity of HELQ to unwind the R-loop thus releasing the RNA for digestion by XRN2. An alternative mechanistic scenario whereby the annealase activity of HELQ is involved in re-annealing the displaced DNA strand, thus removing the RNA from the R-loop, is unlikely as in our *in vitro* assays the displaced sequence of the top strand of our synthetic R-loop substrate is not complementary to the bottom sequence annealed to the RNA. Hence, there is no complementarity to be exploited by the annealase activity of HELQ.

The mechanism of XRN2 recruitment at R-loops is unknown. XRN2 may physically interact with helicases recruited at R-loops. Indeed, previous studies have reported the vital role of RGG/GG motif methylation in DDX5 which was required for physical interaction with XRN2 [52]. Our BioID2 pulldowns also identified DDX5 as a potential HELQ interactant or being near HELQ at R-loops *in vivo*. This could be explained by DDX5 and HELQ sharing redundant R-loop unwinding activities in cells, and XRN2 potentially recruited to R-loops by DDX5 in the first place but could degrade RNAs released from R-loops irrespective of the unwinding helicase. It remains to be established whether XRN2 interacts physically with HELQ at R-loops. Our data are consistent with a physical XRN2-HELQ interaction, but BioID2 does not exclusively detect physical protein–protein interactions since proteins proximal to HELQ at any given time could also be biotinylated and consequently pulled down with this technique. Therefore, XRN2 may interact with HELQ at R-loops or be proximal to HELQ and functionally cooperating without physically interacting with it. Detecting a physical interaction between HELQ and XRN2 may be difficult as such an interaction will be highly dynamic, transient and likely ssDNA dependent. Alternatively, XRN2 may be recruited to R-loops through RPA. The latter has been shown to act as a sensor of R-loops and regulator of RNase H1 [24]. It is plausible that RPA may also regulate XRN2 function at R-loops in a similar manner to RNase H1. Whatever the precise mechanism of XRN2 recruitment at R-loops, our collective data suggest that HELQ and XRN2 cooperate in R-loop resolution.

## Data Availability

The mass spectrometry proteomics data from the BioID2 pulldown experiments have been deposited to the ProteomeXchange Consortium through the PRIDE [55] partner repository with the dataset identifier PXD049246. Supplementary material is available online [56].
